# Cardiac and Renal Protective Effects of Irbesartan via Peroxisome Proliferator‐Activated Receptorγ–Hepatocyte Growth Factor Pathway Independent of Angiotensin II Type 1a Receptor Blockade in Mouse Model of Salt‐Sensitive Hypertension

**DOI:** 10.1161/JAHA.113.000103

**Published:** 2013-04-24

**Authors:** Hiroshi Kusunoki, Yoshiaki Taniyama, Hiromi Rakugi, Ryuichi Morishita

**Affiliations:** 1 Department of Clinical Gene Therapy, Osaka University, Graduate School of Medicine, Suita, Japan (H.K., Y.T., H.R., R.M.); 2 Department of Geriatric Medicine and Nephrology, Osaka University, Graduate School of Medicine, Suita, Japan (H.K., Y.T., H.R., R.M.)

**Keywords:** angiotensin, angiotensin II type 1 receptor blocker, epithelial–mesenchymal transition, hepatocyte growth factor, left ventricular hypertrophy

## Abstract

**Background:**

“Aldosterone breakthrough” observed in patients receiving long‐term treatment with angiotensin blockade is strongly associated with increased risk of left ventricular hypertrophy, poor exercise capacity, refractory proteinuria, and declining glomerular filtration rate through the profibrotic actions of aldosterone. To overcome aldosterone breakthrough, we examined the additional organ‐protective actions of irbesartan, because irbesartan is an angiotensin II type 1 receptor (AT1R) blocker (ARB) with peroxisome proliferator‐activated receptor **(**PPAR)γ agonistic effects, which mediate organ‐protective effects independent of AT1R blockade. In this study, we examined the organ‐protective effects of irbesartan in a salt‐sensitive hypertension model using AT1aR knockout mice.

**Methods and Results:**

Aldosterone and 1% NaCl treatment resulted in a significant increase in severe cardiac and renal fibrosis. Irbesartan, but not losartan, significantly reduced renal fibrosis, glomerular injury through inhibition of macrophage infiltration, epithelial–mesenchymal transition, and oxidative stress. Similarly, cardiac fibrosis and myocyte hypertrophy were decreased by irbesartan, but not losartan, treatment, associated with a significant reduction in oxidative stress. Importantly, anti–hepatocyte growth factor (HGF) neutralizing antibody and a PPARγ antagonist (GW9662) attenuated these organ‐protective effects of irbesartan. HGF protein level was increased by irbesartan, especially in the kidney and heart, while GW9662 treatment inhibited the increase in HGF level.

**Conclusions:**

In this study, we showed that irbesartan, which has not only AT1aR‐blocking effects, but also PPARγ agonistic effects accompanied by HGF expression, inhibited organ damage by aldosterone and salt treatment. Second‐generation ARBs such as irbesartan, which has the dual actions of AT1R blockade and PPARγ activation, may have clinical value for the treatment of hypertensive patients with aldosterone breakthrough.

## Introduction

Angiotensin (Ang) II type 1 receptor (AT1R) blockers (ARBs) have been widely used for the treatment of hypertension and hypertension‐related cardiovascular end‐organ damage.^1^ Interestingly, among the approved ARBs, irbesartan and telmisartan, so‐called “metabosartans,”^2^ were shown to constitute a unique subset of ARBs that are also capable of activating peroxisome proliferator‐activated receptor (PPAR)γ.^3–4^ It is well known that PPARγ agonistic drugs mediate strong organ‐protective effects, including antifibrosis, antioxidative stress, and anti‐inflammatory effects.^5–8^ Thus, the beneficial therapeutic effects of irbesartan in hypertensive patients with metabolic syndrome might be mediated via both AT1R blockade and partial PPARγ agonistic actions.

Recently, hepatocyte growth factor (HGF) has been shown to be a downstream effector of PPARγ agonists.^9^ Our previous studies using HGF transgenic mice (HGF‐Tg) demonstrated that HGF exhibited anti‐inflammatory and antioxidant effects.^10–11^ In particular, the fact that HGF has potent antifibrotic effects in both the heart and kidney through blockade of the profibrotic actions induced by Ang II and transforming growth factor (TGF)‐β1, and stimulation of degradation of fibrosis via matrix metalloproteinase activation is the center of interest.^12–14^ Indeed, another metabosartan, telmisartan, reduced renal fibrosis and inflammation through the PPARγ–HGF pathway, independently of AT1aR blockade, in a unilateral ureteral obstruction model using AT1aR knockout (AT1aR‐KO) mice.^15^ In this study, we further investigated whether irbesartan has specific‐organ protective effects via the PPARγ–HGF pathway independent of AT1aR blockade in a mouse fibrosis model, because, in large clinical trials such as the Irbesartan Microalbuminuria Type 2 Diabetes in Hypertensive Patients (IRMA II) study and the Irbesartan Type II Diabetic Nephropathy Trial (IDNT), irbesartan demonstrated potent renoprotective effects independent of its blood pressure (BP)‐lowering effect.^16–17^ More interestingly, in the Swedish Irbesartan Left Ventricular Hypertrophy Investigation versus Atenolol (SILVHIA) study, irbesartan improved myocardial fibrosis and diastolic function in hypertensive patients with left ventricular hypertrophy.^18–19^ Recently, the Atrial Fibrillation Clopidogrel Trial With Irbesartan for Prevention of Vascular Events (ACTIVE I) study showed that the rate of hospitalization for heart failure was reduced by irbesartan treatment in atrial fibrillation patients.^20^ On the other hand, it has been reported that the aldosterone–mineralocorticoid receptor (MR) pathway is important for the occurrence of cardiovascular events.^21–22^ Blockade of the aldosterone–MR pathway by spironolactone or eplerenone improved the outcome in heart failure or myocardial infarction.^23–24^ Thus, we used salt‐sensitive hypertension mediated by aldosterone and 1% NaCl infusion in AT1aR‐KO mice, as this has been shown to induce severe cardiac fibrosis.^25–26^

## Materials and Methods

### AT1aR‐Deficient Mouse Model of Salt‐Sensitive Hypertension

Eight‐week‐old AT1aR‐KO mice (The Jackson Laboratory) were used for the experiments. Aldosterone (0.15 μg/h; Wako Chemicals) was administered to AT1aR‐KO mice via an osmotic minipump (model 1002; Alzet) for 4 weeks. These mice were maintained on 1% NaCl via drinking water. One week later, AT1aR‐KO mice were pretreated with drugs, according to the established protocol.

Mice were divided into 6 groups: (1) only 1% NaCl treatment, (2) aldosterone (0.15 μg/h)+1% NaCl treatment, (3) aldosterone (0.15 μg/h)+1% NaCl+irbesartan (5 mg/kg per day) treatment, (4) aldosterone (0.15 μg/h)+1% NaCl and losartan (5 mg/kg per day) treatment, (5) aldosterone (0.15 μg/h)+1% NaCl+irbesartan (5 mg/kg per day) and GW9662 (0.5 mg/kg per day) treatment, and (6) aldosterone (0.15 μg/h)+1% NaCl+irbesartan (5 mg/kg per day) and neutralizing HGF‐Ab (200 μg/wk) treatment. Each group contained 5 to 7 mice. Drugs were dissolved in water and administered ad libitum. Mice were housed in the animal facilities of Osaka University with free access to food and water. Mice were killed at 4 weeks after operation, before which BP was measured via the tail‐cuff method.

### Materials

Irbesartan was donated by Shionogi (Osaka, Japan). Losartan was purchased from LKT Laboratories Inc. HGF neutralizing antibody was purchased from Kringle Pharma. All procedures were approved by the Animal Use and Care Committee of Osaka University. GW9662 was purchased from Cayman Chemical Company. Systolic BP and heart rate were measured by the tail‐cuff method (UR‐5000: Softron).

### Immunohistochemical and Dihydroethidium Staining

Immunohistochemical staining was performed on tissues fixed with 4% formalin and embedded in paraffin, as described previously.^15^ Immunostaining of α‐smooth muscle actin (SMA) and F4/80 was performed using mouse antimouse α‐SMA antibody (M0851: DAKO) and mouse antimouse F4/80 antibody (ab6640: Abcam), respectively. Immunostained images were quantified using National Institutes of Health ImageJ software (http://rsb.info.nih.gov/ij/), and then analyzed visually under a light microscope by 2 investigators blinded to treatment. Dihydroethidium staining was performed as previously described.^10–11^

### Evaluation of Glomerular Injury and Renal Fibrosis

Renal sections embedded in paraffin (5 μm thick) were stained with periodic acid–Schiff and examined by light microscopy. As described by Raij et al,^27^glomerular injury scores were graded as 0: 0% to 10%; 1+: 10% to 25%; 2+: 26% to 50%; 3+: 51% to 75%; and 4+: 75% to 100%. They were then analyzed visually under a light microscope by 2 investigators blinded to treatment. To evaluate renal fibrosis, kidney sections (4 or 5 sections, 5 μm thick, per kidney) were stained with Masson's trichrome stain and analyzed visually under a light microscope by 2 investigators blinded to treatment. Masson's trichrome–stained images were subsequently quantified using National Institutes of Health ImageJ software.

### Measurement of HGF

HGF concentration was measured by an enzyme‐linked immunosorbent assay, using an IMMUNIS mouse HGF EIA kit and MMUNIS HGF extraction buffer (Institute of Immunology Co, Ltd). Mouse kidney samples were disintegrated with IMMUNIS HGF extraction buffer using a Multi‐beads shaker (Yasui Kikai) at 2000*g* for 15 seconds. Homogenates were centrifuged at 14 000*g* for 30 minutes. The supernatant was used for HGF assay, according to the manufacturer's instructions.

### Statistical Analysis

Data are expressed as mean±SEM. We performed Wilcoxon rank–sum test as a nonparametric test for the hypothesis of interests, not all the hypotheses. Because sample sizes are small, the *P* values are calculated by the exact method and then adjusted by Hommel's procedure^28^ for multiple comparisons.

## Results

### Antifibrotic Effects of Irbesartan in Kidney of AT1aR‐KO Mice

**Figure 1.**
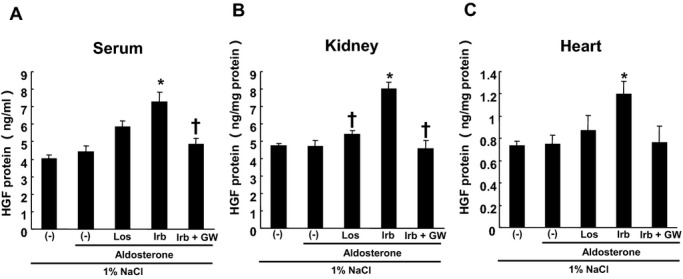
HGF protein concentration in serum, kidney, and heart. A, Serum HGF concentration (ng/mL); B, HGF protein in kidney (ng/mg protein); C, HGF protein in heart (ng/mg protein). *P<0.05 vs 1% NaCl+aldosterone, †*P*<0.05 vs 1% NaCl+aldosterone+irbesartan. Data are shown as mean±SEM (n=5 to 7). HGF‐Ab indicates hepatocyte growth factor neutralizing antibody; (−), nontreated; Los, losartan; Irb, irbesartan; GW, GW9662.

We hypothesized that irbesartan should have additional beneficial effects of organ‐protective actions through the PPARγ–HGF pathway independent of AT1aR blockade. Thus, we first examined in which tissue HGF expression would be increased by irbesartan in AT1aR‐KO mice. As shown in Figure 1, irbesartan significantly increased HGF protein expression in the kidney and heart, in addition to the serum level. This increase in HGF was due to PPARγ activation, independent of AT1aR blockade, because a PPARγ antagonist, GW9662, attenuated the increase. Losartan treatment did not increase local HGF expression in the kidney and heart. Then, we focused on the effects of irbesartan on renal and cardiac changes.

**Figure 2.**
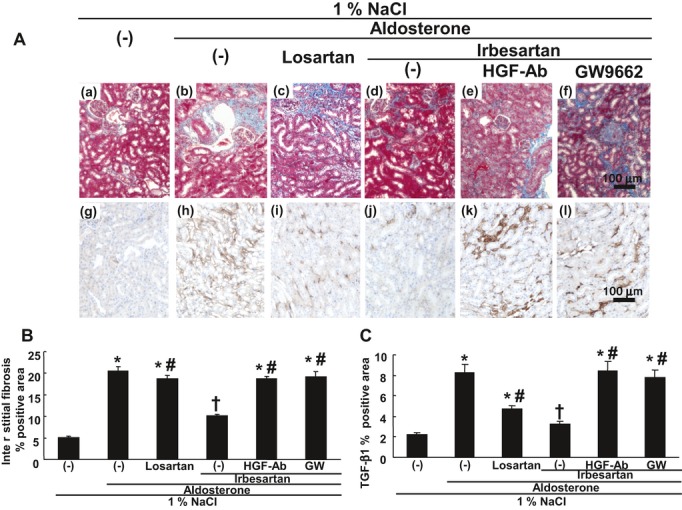
Renal interstitial fibrosis and TGF‐β1–positive area. A, Typical micrographs of kidney with Masson's trichrome staining and TGF‐β1 immunostaining. Upper panels show typical micrographs of kidney with Masson's trichrome staining. Blue color shows fibrotic areas (collagen and fibronectin deposition). (a) Mice treated with only 1% NaCl, (b) mice treated with 1% NaCl+aldosterone (0.15 μg/h), (c) mice treated with 1% NaCl+aldosterone+losartan (10 mg/kg per day), (d) mice treated with 1% NaCl+aldosterone+irbesartan (5 mg/kg per day), (e) mice treated with 1% NaCl+aldosterone+irbesartan+neutralizing HGF‐Ab (200 μg/wk), (f) mice treated with 1% NaCl+aldosterone+irbesartan+GW9662 (PPARγ antagonist, 0.5 mg/kg per day). Bar=100 μm. Lower panels show TGF‐β1 immunostaining in kidneys. Brown color shows TGF‐β1–positive area. (g) Mice treated with only 1% NaCl, (h) mice treated with 1% NaCl+aldosterone (0.15 μg/h), (i) mice treated with 1% NaCl+aldosterone+losartan (10 mg/kg per day), (j) mice treated with 1% NaCl+aldosterone+irbesartan (5 mg/kg per day), (k) mice treated with 1% NaCl+aldosterone+irbesartan+neutralizing HGF‐Ab (200 μg/wk), (l) mice treated with 1% NaCl+aldosterone+irbesartan+GW9662 (PPARγ antagonist, 0.5 mg/kg per day). Bar=100 μm. B, Quantification of interstitial fibrosis percent positive area in kidney. (−), nontreated; HGF‐Ab, HGF neutralizing antibody; GW, GW9662. *P<0.05 vs 1% NaCl alone, †*P*<0.05 vs 1% NaCl+aldosterone, #*P*<0.05 vs 1% NaCl+aldosterone+irbesartan. Data are shown as mean±SEM (n=5 to 7). C, Quantification of TGF‐β 1% positive area in kidney. (−), nontreated; HGF‐Ab, HGF neutralizing antibody; GW, GW9662. *P<0.05 vs 1% NaCl alone, †*P*<0.05 vs 1% NaCl+aldosterone, #*P*<0.05 vs 1% NaCl+aldosterone+irbesartan. Data are shown as mean±SEM (n=5 to 7). TGF‐β1 indicates transforming growth factor‐β1; HGF, hepatocyte growth factor; PPARγ, peroxisome proliferator‐activated receptor‐γ.

Aldosterone infusion and 1% NaCl treatment (Ald‐NaCl treatment) for 1 month resulted in a significant increase in renal fibrosis even in AT1aR‐KO mice (Figure 2A‐b), whereas losartan treatment did not alter renal fibrosis. In contrast, irbesartan treatment significantly reduced fibrosis in the kidney (Figure. 2A‐d). However, the reduction of fibrosis in the kidney by irbesartan was not observed with treatment with GW9662, a PPARγ antagonist, or anti‐HGF neutralizing antibody. These data demonstrated that irbesartan, but not losartan, exhibited antifibrotic effects via the PPARγ–HGF pathway independent of AT1aR blockade. Then, we further explored the molecular mechanisms of the antifibrotic actions of irbesartan.

**Figure 3.**
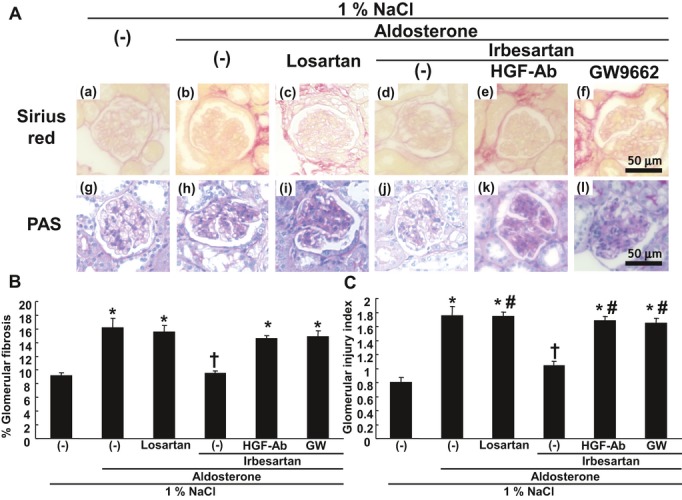
Glomerular fibrosis and glomerular injury. A, Upper panels: typical micrographs of renal glomerular sections with picrosirius red staining to evaluate glomerular fibrosis. (a) Mice treated with only 1% NaCl, (b) mice treated with 1% NaCl+aldosterone (0.15 μg/h), (c) mice treated with 1% NaCl+aldosterone+losartan (10 mg/kg per day), (d) mice treated with 1% NaCl+aldosterone+irbesartan (5 mg/kg per day), (e) mice treated with 1% NaCl+aldosterone+irbesartan+neutralizing HGF‐Ab (200 μg/wk), (f) mice treated with 1% NaCl+aldosterone+irbesartan+GW9662 (PPARγ antagonist, 0.5 mg/kg per day). Bar=50 μm. Lower panels: typical micrographs of renal glomerular sections with periodic acid–Schiff (PAS) staining to evaluate mesangial proliferation. (g) Mice treated with only 1% NaCl, (h) mice treated with 1% NaCl+aldosterone (0.15 μg/h), (i) mice treated with 1% NaCl+aldosterone+losartan (10 mg/kg per day), (j) mice treated with 1% NaCl+aldosterone+irbesartan (5 mg/kg per day), (k) mice treated with 1% NaCl+aldosterone+irbesartan+neutralizing HGF‐Ab (200 μg/wk), (l) mice treated with 1% NaCl+aldosterone+irbesartan+GW9662 (PPARγ antagonist, 0.5 mg/kg per day). Bar=50 μm. B, Quantification of glomerular fibrosis percent positive area. (−), nontreated; HGF‐Ab, HGF neutralizing antibody; GW, GW9662. *P<0.05 vs 1% NaCl alone, †*P*<0.05 vs 1% NaCl+aldosterone. Data are shown as mean±SEM (n=5 to 7). C, Quantification of glomerular injury. (−), nontreated; HGF‐Ab, HGF neutralizing antibody; GW, GW9662. *P<0.05 vs 1% NaCl alone, †*P*<0.05 vs 1% NaCl+aldosterone, #*P*<0.05 vs 1% NaCl+aldosterone+irbesartan. Data are shown as mean±SEM (n=5 to 7). HGF indicates hepatocyte growth factor; PPARγ, peroxisome proliferator‐activated receptor‐γ.

With the use of immunohistochemical staining of TGF‐β1 (a fibrogenic cytokine), irbesartan treatment significantly decreased TGF‐β1 expression in the tubulointerstitial area of the kidney (Figure 2A‐j). In contrast, losartan only slightly suppressed TGF‐β1 expression. The decrease in TGF‐β1 expression in the tubulointerstitial area in the kidney by irbesartan was significantly attenuated by GW9662 or anti‐HGF neutralizing antibody. Of importance, aldosterone mediated not only interstitial fibrosis but also mesangial expansion in the glomerulus and fibrosis around the glomerulus in this model. Thus, we next evaluated glomerular injury in the kidney. Severe fibrosis around the glomerulus could be detected by Sirius red staining (Figure 3A‐b). Also, mesangial expansion in the glomerulus by Ald‐NaCl treatment was detected. Similarly, irbesartan, but not losartan, significantly improved glomerular fibrosis and injury (Figure 3), whereas treatment with GW9662 or anti‐HGF neutralizing antibody reversed these beneficial effects of irbesartan.

**Figure 4.**
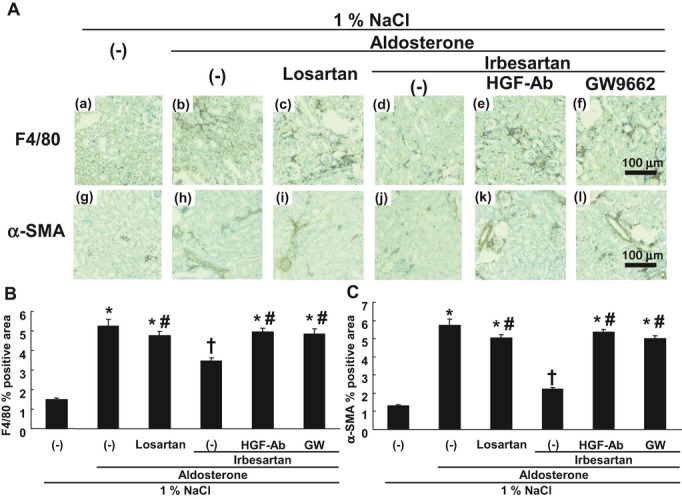
Immunohistochemical staining for F4/80 and α‐SMA. A, Upper panels: typical micrographs of kidney with immunohistochemical staining for F4/80. (a) Mice treated with only 1% NaCl, (b) mice treated with 1% NaCl+aldosterone (0.15 μg/h), (c) mice treated with 1% NaCl+aldosterone+losartan (10 mg/kg per day), (d) mice treated with 1% NaCl+aldosterone+irbesartan (5 mg/kg per day), (e) mice treated with 1% NaCl+aldosterone+irbesartan+neutralizing HGF‐Ab (200 μg/wk), and (f) mice treated with 1% NaCl+aldosterone+irbesartan+GW9662 (PPARγ antagonist, 0.5 mg/kg per day). Bar=100 μm. Lower panels: typical micrographs of kidney with immunohistochemical staining for α‐SMA. (g) Mice treated with only 1% NaCl, (h) mice treated with 1% NaCl+aldosterone (0.15 μg/h), (i) mice treated with 1% NaCl+aldosterone+losartan (10 mg/kg per day), (j) mice treated with 1% NaCl+aldosterone+irbesartan (5 mg/kg per day), (k) mice treated with 1% NaCl+aldosterone+irbesartan+neutralizing HGF‐Ab (200 μg/wk), (l) mice treated with 1% NaCl+aldosterone+irbesartan+GW9662 (PPARγ antagonist, 0.5 mg/kg/day). Bar=100 μm. B, Quantification of F4/80% positive area in kidney. (−), nontreated; HGF‐Ab, HGF neutralizing antibody; GW, GW9662. *P<0.05 vs 1% NaCl alone, †*P*<0.05 vs 1% NaCl+aldosterone, #*P*<0.05 vs 1% NaCl+aldosterone+irbesartan. Data are shown as mean±SEM (n=5 to 7). C, Quantification of α‐SMA percent positive area in kidney. (−), nontreated; HGF‐Ab, HGF neutralizing antibody; GW, GW9662. *P<0.05 vs 1% NaCl alone, †*P*<0.05 vs 1% NaCl+aldosterone, #*P*<0.05 vs 1% NaCl+aldosterone+irbesartan. Data are shown as mean±SEM (n=5 to 7). α‐SMA indicates α‐smooth muscle actin; HGF, hepatocyte growth factor; PPARγ, peroxisome proliferator‐activated receptor‐γ.

Macrophage infiltration and epithelial–mesenchymal transition (EMT) were detected in renal interstitial tissue. It has been reported that aldosterone stimulates inflammatory cells such as macrophages, which play crucial rules in renal fibrosis and oxidative stress.^29^ Mineralocorticoid receptor activation of macrophages may play a major role in aldosterone‐induced inflammation. Of importance, irbesartan treatment significantly reduced the macrophage infiltration area in the kidney as detected with F4/80 immunostaining (Figure 4A‐a through f), whereas treatment with GW9662 or anti‐HGF neutralizing antibody attenuated this beneficial effect of irbesartan. Irbesartan also significantly reduced myofibroblast area as detected with α‐SMA immunostaining in the kidney (Figure 4A‐g through l), whereas treatment with GW9662 or anti‐HGF neutralizing antibody attenuated the reduction in myofibroblast area by irbesartan.

**Figure 5.**
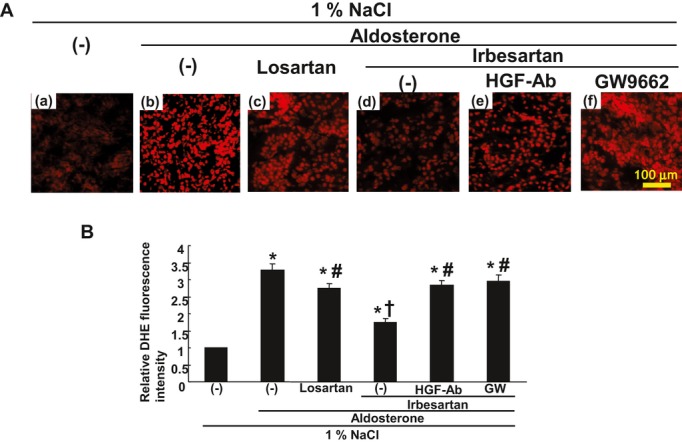
Dihydrothidium (DHE) staining in kidney. A, Typical micrographs of kidney with DHE staining. (a) Mice treated with only 1% NaCl, (b) mice treated with 1% NaCl+aldosterone (0.15 μg/h), (c) mice treated with 1% NaCl+aldosterone+losartan (10 mg/kg per day), (d) mice treated with 1% NaCl+aldosterone+irbesartan (5 mg/kg per day), (e) mice treated with 1% NaCl+aldosterone+irbesartan+neutralizing HGF‐Ab (200 μg/wk), and (f) mice treated with 1% NaCl+aldosterone+irbesartan+GW9662 (PPARγ antagonist, 0.5 mg/kg per day). Bar=100 μm. B, Quantification of fluorescence intensity of DHE staining visualized by confocal microscopy (%) in kidney. (−), nontreated; HGF‐Ab, HGF neutralizing antibody; GW, GW9662. *P<0.05 vs 1% NaCl alone, †*P*<0.05 vs 1% NaCl+aldosterone, #*P*<0.05 vs 1% NaCl+aldosterone+irbesartan. Data are shown as mean±SEM (n=5 to 7). HGF indicates hepatocyte growth factor; PPARγ, peroxisome proliferator‐activated receptor‐γ.

Numerous studies have focused on the role of oxidative stress and inflammation in the initiation of aldosterone‐mediated organ damage. Indeed, a significant increase in oxidative stress was detected in the kidney of this model as assessed with DHE staining (Figure 5). Because our previous studies demonstrated that HGF showed potent antioxidative actions, we further examined the antioxidative effects of irbesartan. As expected, irbesartan, but not losartan, treatment significantly suppressed redox signaling detected by DHE fluorescence. In contrast, treatment with GW9662 or anti‐HGF neutralizing antibody reversed this beneficial effect of irbesartan (Figure 5).

### Antifibrotic Effects of Irbesartan in Hearts of AT1aR KO Mice

**Figure 6.**
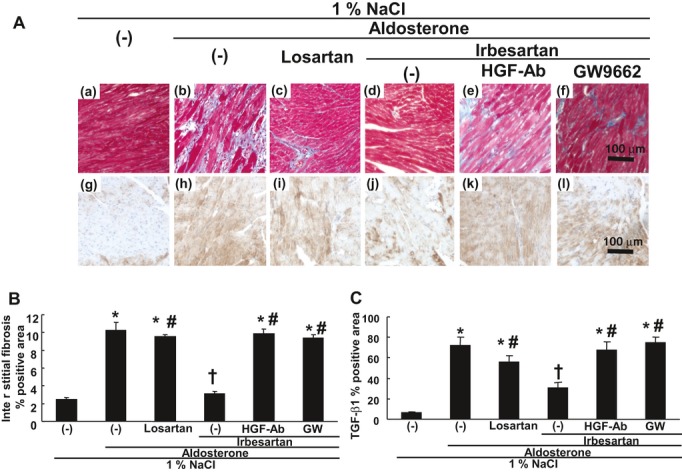
Cardiac interstitial fibrosis and TGF‐β1–positive area. A, Typical micrographs of heart with Masson's trichrome staining and TGF‐β1 immunostaining. Upper panels show typical micrographs of heart with Masson's trichrome staining. Blue color shows fibrotic areas (collagen and fibronectin deposition); (a) mice treated only 1% NaCl, (b) mice treated 1% NaCl+aldosterone (0.15 μg/h), (c) mice treated 1% NaCl+aldosterone+losartan (10 mg/kg per day), (d) mice treated 1% NaCl+aldosterone (0.15 μg/h)+irbesartan (5 mg/kg per day), (e) mice treated 1% NaCl+aldosterone+irbesartan+neutralizing HGF‐Ab (200 μg/wk), (f) mice treated 1% NaCl+aldosterone+irbesartan+GW9662 (PPARγ antagonist, 0.5 mg/kg per day). Bar=100 μm. Lower panels show TGF‐β1 immunostaining in hearts. Brown color shows TGF‐β1–positive area. (g) Mice treated with only 1% NaCl, (h) mice treated with 1% NaCl+aldosterone (0.15 μg/h), (i) mice treated with 1% NaCl+aldosterone+losartan (10 mg/kg per day), (j) mice treated with 1% NaCl+aldosterone+irbesartan (5 mg/kg per day), (k) mice treated with 1% NaCl+aldosterone+irbesartan+neutralizing HGF‐Ab (200 μg/wk), and (l) mice treated with 1% NaCl+aldosterone+irbesartan+GW9662 (PPARγ antagonist, 0.5 mg/kg per day). Bar=100 μm. B, Quantification of interstitial fibrosis percent positive area in heart. (−), nontreated; HGF‐Ab, HGF neutralizing antibody; GW, GW9662. *P<0.05 vs 1% NaCl alone, †*P*<0.05 vs 1% NaCl+aldosterone, #*P*<0.05 vs 1% NaCl+aldosterone+irbesartan. Data are shown as mean±SEM (n=5 to 7). C, Quantification of TGF‐β 1% positive area in heart. (−), nontreated; HGF‐Ab, HGF neutralizing antibody; GW, GW9662. *P<0.05 vs 1% NaCl alone, †*P*<0.05 vs 1% NaCl+aldosterone, #*P*<0.05 vs 1% NaCl+aldosterone+irbesartan. Data are shown as mean±SEM (n=5 to 7). TGF‐β1 indicates transforming growth factor‐β1; HGF, hepatocyte growth factor; PPARγ, peroxisome proliferator‐activated receptor‐γ.

In this model, similar fibrotic lesions were also found in the heart. Similar to their effect on renal fibrosis, irbesartan, but not losartan, treatment significantly reduced fibrosis in the heart (Figure 6), whereas the reduction of fibrosis in the heart by irbesartan was also attenuated by treatment with GW9662, a PPARγ antagonist, or anti‐HGF neutralizing antibody. Even in the interstitial area of the heart, irbesartan treatment significantly decreased TGF‐β1 expression (Figure 6). In contrast, losartan only slightly suppressed TGF‐β1 expression. The decrease in TGF‐β1 expression in the heart by irbesartan was significantly attenuated by GW9662 or anti‐HGF neutralizing antibody.

**Figure 7.**
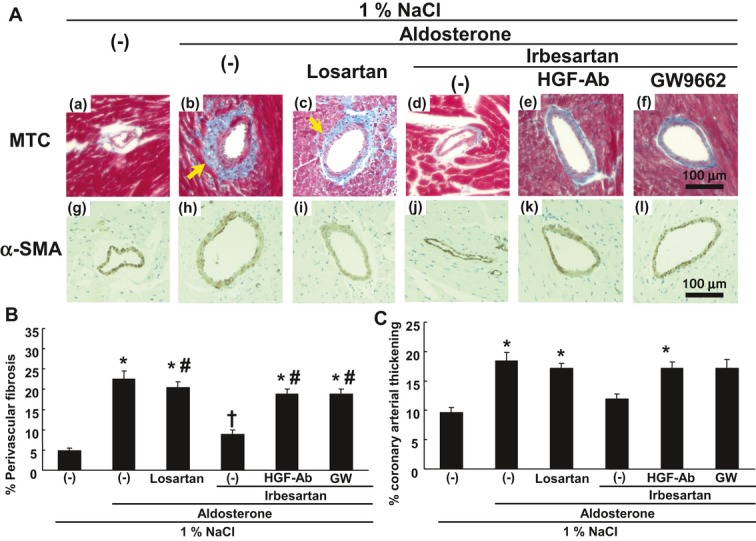
Perivascular fibrosis and proliferation of vascular smooth muscle cells (VSMC) around coronary artery. A, Upper panels: typical micrographs of coronary artery in heart with Masson's trichrome staining (MTC). Blue color shows fibrotic areas (collagen and fibronectin deposition). Lower panels show micrographs of coronary artery in heart with α‐SMA immunohistchemical staining. (a, g) Mice treated with only 1% NaCl, (b, h) mice treated 1% NaCl+aldosterone (0.15 μg/h), (c, i) mice treated with 1% NaCl+aldosterone+losartan (10 mg/kg per day), (d, j) mice treated with 1% NaCl+aldosterone+irbesartan (5 mg/kg per day), (e, k) mice treated with 1% NaCl+aldosterone+irbesartan (5 mg/kg per day)+neutralizing HGF‐Ab (200 μg/wk), and (f, l) mice treated with 1% NaCl+aldosterone+irbesartan+GW9662 (PPARγ antagonist, 0.5 mg/kg per day). Bar=100 μm. B, Quantification of perivascular fibrosis percent positive area around coronary artery. (−), nontreated; HGF‐Ab, HGF neutralizing antibody; GW, GW9662. *P<0.05 vs 1% NaCl alone, †*P*<0.05 vs 1% NaCl+aldosterone, #*P*<0.05 vs 1% NaCl+aldosterone+irbesartan. Data are shown as mean±SEM (n=5 to 7). C, Quantification of coronary arterial thickening (%).(−), nontreated; HGF‐Ab, HGF neutralizing antibody; GW, GW9662. *P<0.05 vs 1% NaCl alone. Data are shown as mean±SEM (n=5 to 7). α‐SMA indicates α‐smooth muscle actin; HGF, hepatocyte growth factor; PPARγ, peroxisome proliferator‐activated receptor‐γ.

**Figure 8.**
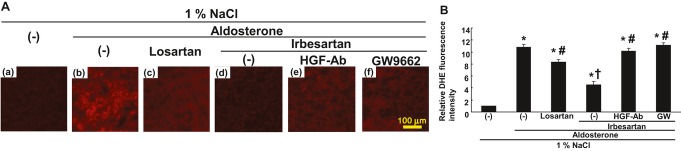
Dihydrothidium (DHE) staining in heart. A, Typical micrographs of heart with DHE staining. (a) Mice treated with only 1% NaCl, (b) mice treated with 1% NaCl+aldosterone (0.15 μg/h), (c) mice treated with 1% NaCl+aldosterone+losartan (10 mg/kg per day), (d) mice treated with 1% NaCl+aldosterone+irbesartan (5 mg/kg per day), (e) mice treated with 1% NaCl+aldosterone+irbesartan+neutralizing HGF‐Ab (200 μg/wk), and (f) mice treated with 1% NaCl+aldosterone+irbesartan+GW9662 (PPARγ antagonist, 0.5 mg/kg per day). Bar=100 μm. B, Quantification of fluorescence intensity of DHE staining visualized by confocal microscopy (%) in heart. (−), nontreated; HGF‐Ab, HGF neutralizing antibody; GW, GW9662. *P<0.05 vs 1% NaCl alone, †*P*<0.05 vs 1% NaCl+aldosterone, #*P*<0.05 vs 1% NaCl+aldosterone+irbesartan. Data are shown as mean±SEM (n=5 to 7). HGF indicates hepatocyte growth factor; PPARγ, peroxisome proliferator‐activated receptor‐γ.

As our recent report demonstrated that HGF reduced cardiac fibrosis by the inhibition of EMT in a transverse aortic constriction (TAC) model in HGF‐Tg mice, we also examined α‐SMA–positive myofibroblasts in the coronary artery. Consistent with the previous report, aldosterone and salt treatment provoked severe fibrosis around the coronary artery and coronary artery thickening (Figure 7). In addition, α‐SMA–positive myofibroblast proliferation was promoted around the coronary artery by Ald‐NaCl treatment. Interestingly, severe fibrosis around the coronary artery and coronary artery thickening was also reduced by irbesartan treatment, whereas losartan had no effect (Figure 7A‐a through f). Similarly, α‐SMA–positive myofibroblast proliferation was decreased by irbesartan but not by losartan (Figure 7A‐g through l). Treatment with GW9662 or anti‐HGF neutralizing antibody attenuated these changes by irbesartan (Figure 7). Expression of reactive oxygen species detected with DHE staining was also increased in the heart by Ald‐NaCl treatment (Figure 8). As expected, irbesartan, but not losartan, significantly suppressed redox signaling detected by DHE fluorescence, whereas treatment with GW9662 or anti‐HGF neutralizing antibody attenuated the decrease in oxidative stress by irbesartan (Figure 8).

**Figure 9.**
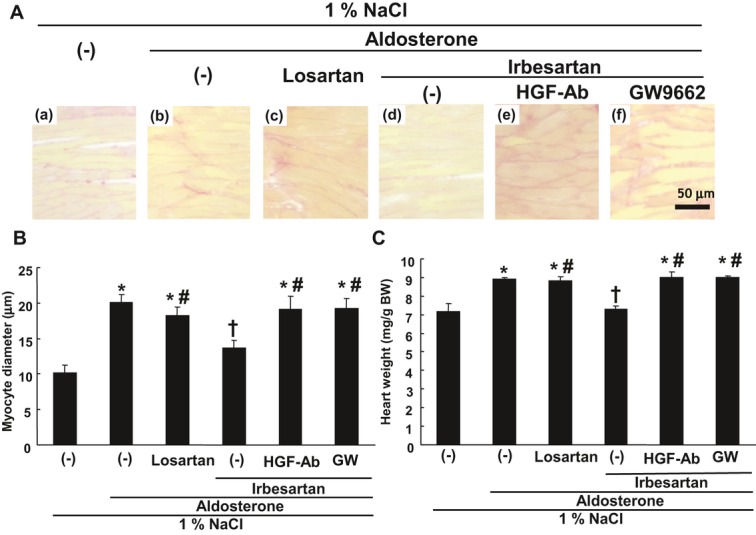
Cardiac myocyte diameter and heart weight. A, Typical micrographs of heart with picrosirius red staining to evaluate cardiac myocyte diameter. (a) Mice treated with only 1% NaCl, (b) mice treated with 1% NaCl+aldosterone (0.15 μg/h), (c) mice treated with 1% NaCl+aldosterone+losartan (10 mg/kg per day), (d) mice with treated 1% NaCl+aldosterone+irbesartan (5 mg/kg per day), (e) mice treated with 1% NaCl+aldosterone+irbesartan+neutralizing HGF‐Ab (200 μg/wk), and (f) mice treated with 1% NaCl+aldosterone+irbesartan+GW9662 (PPARγ antagonist, 0.5 mg/kg per day). Bar=50 μm. B, Quantification of cardiac myocyte diameter. (−), nontreated; HGF‐Ab, HGF neutralizing antibody; GW, GW9662. *P<0.05 vs 1% NaCl alone, †*P*<0.05 vs 1% NaCl+aldosterone, #*P*<0.05 vs 1% NaCl+aldosterone+irbesartan. Data are shown as mean±SEM (n=5 to 7). C, Heart weight (mg/g body weight). (−), nontreated; HGF‐Ab, HGF neutralizing antibody; GW, GW9662. *P<0.05 vs 1% NaCl alone, †*P*<0.05 vs 1% NaCl+aldosterone, #*P*<0.05 vs 1% NaCl+aldosterone+irbesartan. Data are shown as mean±SEM (n=5 to 7). HGF indicates hepatocyte growth factor; PPARγ, peroxisome proliferator‐activated receptor‐γ.

Finally, we examined whether irbesartan reduced the hypertrophy of cardiac fibers and increase in heart weight induced by aldosterone without AT1aR. Of importance, irbesartan, but not losartan, reduced the hypertrophy of cardiac fibers and heart weight (Figure 9). Consistently, treatment with GW9662 or anti‐HGF neutralizing antibody attenuated these changes by irbesartan (Figure 9). Both the renal and cardiac protective effects of irbesartan were not due to BP lowering, because this dose of irbesartan did not lower BP (Table 1).

**Table 1.** Blood Pressure Before and After Treatment

	After	Before
NaCl alone	84.4±1.3/54.7±1.6	89.9±1.7/57.6±1.8
Ald+NaCl	84.1±1.6/55.9±1.5	123.3±2.1*/80.1±0.9*
Ald+NaCl+irbesartan	83.2±0.7/54.4±1.6	121.4±0.9*/78.2±2.4*
Ald+NaCl+losartan	85.4±0.8/56.3±1.7	122.2±1.0*/74.8±1.3*
Ald+NaCl+irbesartan+GW9662	85.2±1.2/53.4±1.3	121.0±1.0*/74.4±1.3*
Ald+NaCl+irbesartan+neutralizing HGF‐Ab	84.8±2.4/56.7±1.5	122.6±1.2*/76.3±2.3*

Data are mean±SEM. We compared the blood pressure of NaCl alone with that of other groups after Ald‐NaCl treatment. It means there was no difference in blood pressure before Ald‐NaCl treatment in all groups, but after the treatment blood pressure increases were observed in all groups treated with aldosterone and NaCl. On the other hand, there were no blood pressure increases in the group treated only with NaCl (n=5 to 7). Ald indicates aldosterone; HGF, hepatocyte growth factor.

* *P*<0.05 vs 1% NaCl alone.

## Discussion

The present study demonstrated that irbesartan, a second‐generation ARB metabosartan, reduced renal and cardiac fibrosis induced by aldosterone treatment, a model of salt‐sensitive hypertension, in AT1aR KO mice. These data clearly demonstrated that irbesartan exhibited organ‐protective actions independent of Ang II receptor blockade, probably due to inhibition of inflammation and EMT and a decrease in oxidative stress. Importantly, these additive organ‐protective actions of irbesartan, in addition to Ang II blockade, might be due to PPARγ‐induced local HGF expression, because a PPARγ antagonist or anti‐HGF neutralizing antibody attenuated the improvement of renal and cardiac fibrosis by irbesartan.

The present study raises several important points. First, this salt‐sensitive hypertension model in AT1aR KO mice is an “aldosterone breakthrough” or “aldosterone escape” model. It is well known that the plasma aldosterone level is increased in 30% to 40% of patients receiving long‐term angiotensin‐converting enzyme inhibitors and/or ARBs. This “aldosterone breakthrough” would have important clinical consequences through the profibrotic actions of aldosterone. Indeed, previous studies indicated that “aldosterone breakthrough” is strongly associated with increased risk of left ventricular hypertrophy, poor exercise capacity, refractory proteinuria, and declining glomerular filtration rate. Moreover, aldosterone and salt synergically promoted organ damage in the heart and kidney.^25^ The present study demonstrated that irbesartan prevented renal and cardiac organ damage even in a condition of “aldosterone breakthrough.” These findings suggest possible new clinical benefits of using irbesartan to treat such fibrotic conditions in patients treated with ARBs.

Second, although activation of PPARγ is well known to have organ‐protective actions such as reduction of cardiovascular events and proteinuria and delaying the progression of diabetic nephropathy, the mediator of PPARγ is still unclear. The present study showed HGF to be a potent downstream effector of PPARγ. HGF is a well‐known antifibrotic and antioxidative growth factor.^30–31^ In contrast, TGF‐β1 is a mediator of the profibrotic effects of aldosterone, such as the differentiation and proliferation of fibroblasts and collagen deposition, as aldosterone increased TGF‐β1 mRNA expression and signaling in various cells including cultured cardiomyocytes^32^ and animal models.^33–34^ Consistent with our previous report, irbesartan, a PPARγ agonistic ARB, upregulated HGF protein levels in serum, kidney, and heart. In contrast, irbesartan reduced TGF‐β1 expression in the kidney and heart. The contribution of the PPARγ–HGF pathway was confirmed by the observation that the reduction in TGF‐β1 expression in the kidney and heart by irbesartan was diminished by either anti‐HGF neutralizing antibody or GW9662.

Third, the organ‐protective actions of the PPARγ–HGF pathway are widely attributed to various actions such as inhibition of inflammation, inhibition of EMT, and decrease of oxidative stress. Although it is already reported that aldosterone induced EMT via oxidative stress of mitochondrial origin,^35^ activation of HGF clearly suppressed EMT in both the kidney and the heart in the present study. In addition, the potent antioxidative effect of HGF was shown in this study. Many studies have focused on the role of oxidative stress and inflammation in the initiation of aldosterone‐mediated end organ damage. It is important that HGF inhibited pathways involved in the progression from inflammation to remodeling and fibrosis. Macrophage infiltration also plays a crucial rule in inflammation, fibrosis, and oxidative stress in the kidney and heart.^29,36–38^ The present study showed that irbesartan reduced macrophage infiltration in the kidney and heart via the PPARγ–HGF pathway in an AT1aR KO model.

There are some limitations of this study. First, the mice used in this study were AT1aR‐deficient mice. Thus, the additive protective effects by irbesartan might not be due to PPARγ activation. It is possible that the additive kidney and heart protective effects by irbesartan might be related to AT1b receptor blockade. Second, another potential limitation of this study is the use of the tail‐cuff technique to measure BP. The tail‐cuff technique does not give 24‐hour BP measurements and BP variability. Thus, it is possible that some of the greater renoprotective effects of irbesartan versus losartan might have been due to the greater effects of irbesartan on 24‐hour BP or other components of BP not measured by the tail‐cuff technique. In IRMA2 and IDNT, irbesartan demonstrated potent renoprotective effects independent of its BP‐lowering effect. However, because IRMA2 and IDNT did not include continuous 24‐hour measurements of BP, these studies did not prove that the greater benefits afforded by irbesartan were truly independent of BP‐lowering effects. Indeed, Griffin et al mentioned the limitation of the tail‐cuff methodology, which provides only intermittent BP data. They performed BP radiotelemetry using stroke‐prone spontaneously hypertensive rats (SHRSP) and concluded that renal protection by rennin‐angiotensin system blockade is dependent on a continuous BP‐lowering effect. Further basic and clinical investigation is needed to prove how BP‐lowering effects contribute the organ protective effects of ARBs by referencing not only intermittent BP data but also continuous BP data.^39^

**Figure 10.**
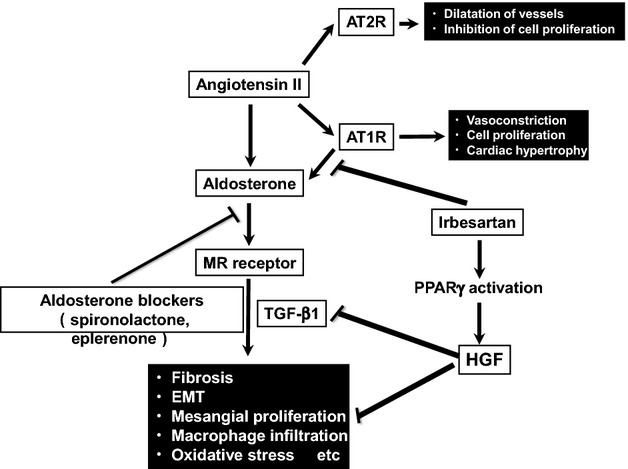
Summary of this study. Irbesartan suppresses fibrosis, EMT, mesangial proliferation, macrophage infiltration, and oxidative stress, inducing HGF expression through its PPARγ‐agonistic effects, independent of AT1R‐blocking activity. EMT indicates epithelial–mesenchymal transition; HGF, hepatocyte growth factor; PPARγ, peroxisome proliferator‐activated receptor‐γ; MR, mineralocorticoid receptor; TGF‐β1 indicates transforming growth factor‐β1; AT1R, angiotensin II type 1 receptor; AT2R, angiotensin II type 2 receptor.

In this study, we showed that irbesartan, which has not only AT1aR‐blocking effects but also PPARγ‐agonistic effects accompanied by HGF expression, inhibited the organ damage by aldosterone and salt treatment. Many basic and clinical studies showed that MR antagonists like spironolactone or eplerenone exhibit organ‐protective effects by inhibiting the signal pathway from MR directly. It has been believed that ARBs inhibit the MR pathway indirectly by inhibiting the pathway from AT1aR. However, the present study showed that there is another pathway in the organ‐protective effects of irbesartan through PPARγ agonistic effects. Irbesartan showed antifibrotic, anti‐inflammatory, and antioxidative effects and attenuated aldosterone/MR signaling via the PPARγ–HGF pathway independent of AT1aR signaling (Figure 10). “Aldosterone breakthrough” is one of the unresolved cardiovascular risks, since inhibition of the rennin‐angiotensin system does not control all pathophysiological mechanisms of hypertension or cardiovascular risk. Second‐generation ARBs such as irbesartan, which has the dual actions of AT1R blockade and PPARγ activation, may have clinical value in treating such patients. Further development of the next generation of ARBs with added organ‐protective actions that can target additional mechanisms of hypertension, cardiovascular disease, and diabetes might be important.
